# Counteraction of inflammatory activity in CAA-related subarachnoid hemorrhage

**DOI:** 10.1007/s00415-022-11437-9

**Published:** 2022-10-29

**Authors:** Stefanie Schreiber, Anna-Charlotte John, Cornelius J. Werner, Stefan Vielhaber, Hans-Jochen Heinze, Oliver Speck, Jens Würfel, Daniel Behme, Hendrik Mattern

**Affiliations:** 1 Department of Neurology, Otto von Guericke University, Leipziger Strasse 44, 39120 Magdeburg, Germany; 2 German Center for Neurodegenerative Diseases (DZNE), Magdeburg, Germany; 3 Center for Behavioral Brain Sciences (CBBS), Magdeburg, Germany; 4 University Clinic for Neuroradiology, Otto von Guericke University, Magdeburg, Germany; 5 Department of Neurology and Geriatrics, Johanniter Hospital, Stendal, Germany; 6 Department of Neurology, Medical Faculty, RWTH Aachen University, Aachen, Germany; 7 Leibniz Institute for Neurobiology (LIN), Magdeburg, Germany; 8 Department of Biomedical Magnetic Resonance (BMMR), Otto von Guericke University, Magdeburg, Germany; 9 Medical Image Analysis Center (MIAC), Basel, Switzerland

## Dear Prof. Roger A. Barker, Prof. Massimo Filippi, Prof. Michael Strupp,

spontaneous convexal subarachnoid hemorrhage (cSAH) is a marker of cerebral amyloid angiopathy (CAA) in the elderly that indicates poor disease outcome through its predictive value for future intracerebral hemorrhage (ICH) which is a significant cause for morbidity and mortality in CAA patients [1]. Subarachnoid hemorrhage results from leptomeningeal vessel leakage, commonly related to advanced CAA severity and recurrent intrasulcal bleeding, which precedes cortical superficial siderosis (cSS) development (the chronic variant of cSAH) that itself is the strongest independent predictor for future CAA-related ICH [2, 3].

Currently, there is no causal therapy preventing ongoing recurrent vessel leakage and risk reduction of associated ICH is just centered around the (non-causal) management of comorbidities, i.e., mainly long-term blood pressure control and individual decision-making regarding the need for initiation and continuation of anticoagulation [4].

We here demonstrate the case of a 75-year old female diagnosed with probable CAA according to the Boston Criteria version 2.0 due to recurrent cSAH, subsequent multifocal cSS development and corresponding clinical presentation of acute headache and transient focal neurological episodes during intrasulcal bleeding events [5].

**Fig. 1:**
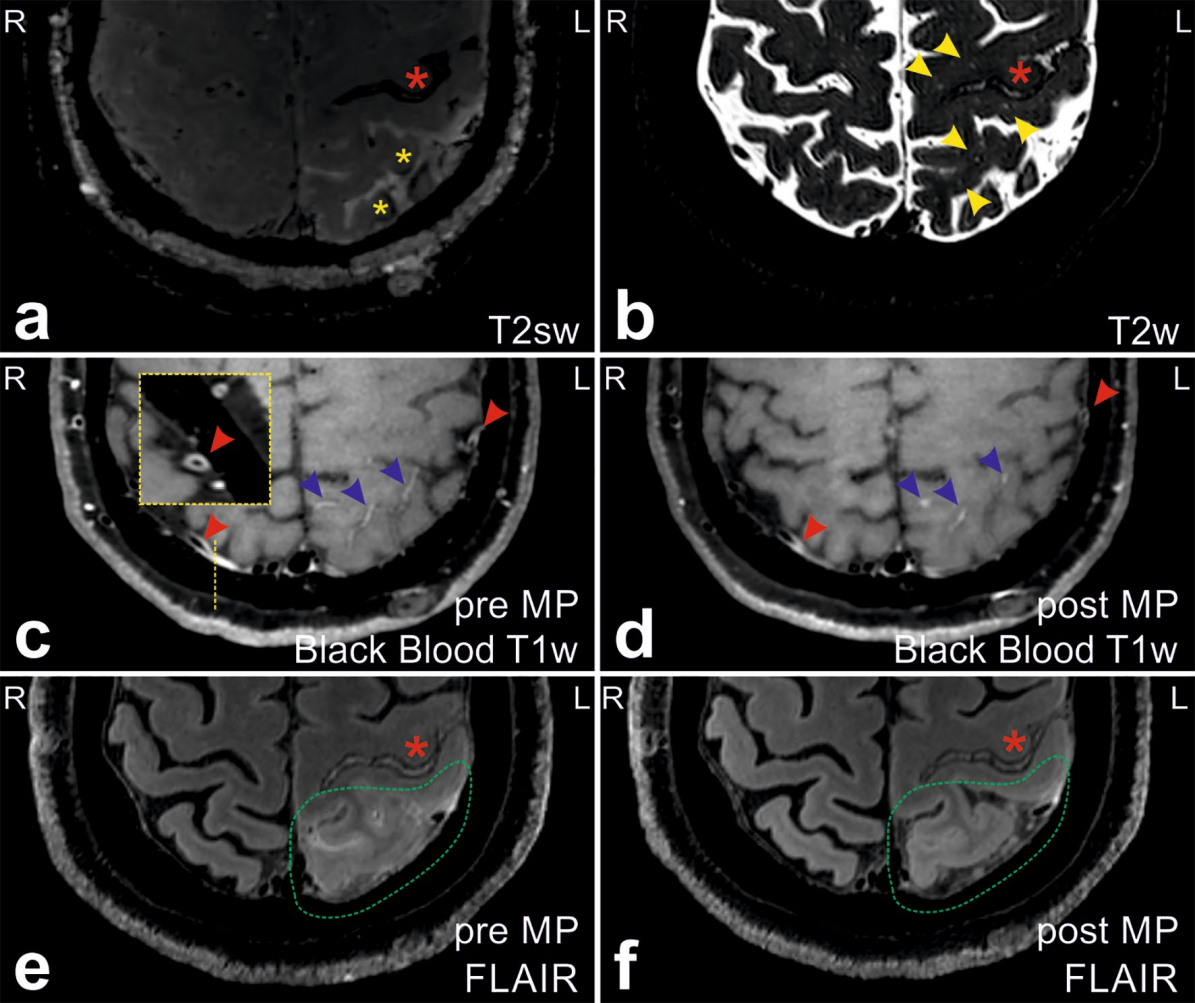
In proximity to previous cSS (red asterisk), new cSS (yellow asterisks) evolved after spontaneous convexal subarachnoid hemorrhage (cSAH) in the parieto-occipital cerebral cortex of a 75-year old female CAA patient (**a**). In the surrounding brain parenchyma, multiple enlarged perivascular spaces (yellow arrowheads) (**b**), meningeal arterial wall (red arrowheads; sagittal cross-section along yellow dashed line), sulcal venous contrast enhancement (blue arrowheads) (**c**), and multifocal (peri-)sulcal edema (framed in green) (**e**) became evident (see also Supplemental Video). Vessel wall contrast-enhancement and edema regressed after systemic methyl prednisolone (MP) application (**d**, **f**)

We had the chance to scan the patient at both, 3 Tesla (T) and 7 T magnetic resonance imaging (MRI), two months after the latest cSAH. Black-blood T1-weighted and postcontrast fluid-attenuated inversion recovery (FLAIR) 3 T MRI sequences depicted leptomeningeal arterial and sulcal venous vessel wall enhancement together with (peri)sulcal cortical edema, which were in the direct proximity of cSS newly evolving from previous cSAH, but vessel wall enhancement became also evident in the contralateral hemisphere (Fig. 1a, c, e). These findings were accompanied by pronounced perivascular space enlargement in the ipsilateral hemisphere (Fig. 1b). Two hours after contrast administration, the 7 T MRI (whole brain, 0.7 mm isotropic resolution) depicted small hyperintense features within the straight sagittal sinus and extended leakage of contrast agent into the sulci around the cSS compared to the 3 T (see Supplemental Video), indicating a potential interaction between blood–brain barrier (BBB) breakdown, impaired clearance and CAA.

Imaging findings resemble the results derived from a larger CAA cohort demonstrating extravasation of gadolinium in postcontrast T1 images at the location of cSAH/cSS together with more widespread leptomeningeal enhancement not restricted to the cSAH/cSS site and accompanied by focal cortical swelling in the vicinity of the intrasulcal hemorrhage [2].

In CAA-related inflammation (CAA-ri), (widespread) leptomeningeal enhancement is a common MRI finding as well, pointing toward similar mechanisms of wall leakage, BBB breakdown and perivascular peripheral and neuroinflammatory activity surround amyloid-laden vessels [6, 7].

We therefore decided to counteract high CAA disease and suspected inflammatory activity in the presented patient through systemic high-dose methyl prednisolone (MP), 1 g daily for 3 days, which was initiated immediately after MRI; MP is frequently applied in CAA-ri, but so far not in clinical use for the more common non-inflammatory forms of CAA [7].

Repeated 3 T MRI, conducted 3 days after MP termination, displayed successful partial recovery of vessel leakage and cortical edema (Fig. 1d, f).

After therapy we had the chance to follow the patient for a monthly clinical and 3 T MRI follow-up over a timespan of 9 months. No new CAA-related symptoms occurred and no cSAH or newly evolving cSS or ICH became evident in the repeated MRI scans.

Based on the presented findings, we recommend a systematic MRI study applying contrast-enhanced imaging in patients with cSAH and/or cSS so far classified as non-inflammatory CAA variants. The aim would be to explore the generalizability of these single-case results of pronounced inflammatory activity in these patients with advanced CAA severity. If provable, this would open new perspectives not only on innovative MRI protocols needed to better stratify this vulnerable subcohort in-vivo, but also for targeted and causal immunosuppressive treatment with the chance to reduce these patients' high risk for future ICH.

## Supplementary Information

Below is the link to the electronic supplementary material.

Supplementary file1 (AVI 5087 KB)
